# Ion Channels in Obesity: Pathophysiology and Potential Therapeutic Targets

**DOI:** 10.3389/fphar.2016.00058

**Published:** 2016-03-30

**Authors:** Luiz H. C. Vasconcelos, Iara L. L. Souza, Lílian S. Pinheiro, Bagnólia A. Silva

**Affiliations:** ^1^Laboratório de Farmacologia Funcional Prof. George Thomas, Programa de Pós-graduação em Produtos Naturais e Sintéticos Bioativos, Centro de Ciências da Saúde, Universidade Federal da ParaíbaJoão Pessoa, Brazil; ^2^Departamento de Ciências Farmacêuticas, Centro de Ciências da Saúde, Universidade Federal da ParaíbaJoão Pessoa, Brazil

**Keywords:** energetic metabolism, food intake, adipose cells, ion channels, obesity

## Abstract

Obesity is a multifactorial disease related to metabolic disorders and associated with genetic determinants. Currently, ion channels activity has been linked to many of these disorders, in addition to the central regulation of food intake, energetic balance, hormone release and response, as well as the adipocyte cell proliferation. Therefore, the objective of this work is to review the current knowledge about the influence of ion channels in obesity development. This review used different sources of literature (Google Scholar, PubMed, Scopus, and Web of Science) to assess the role of ion channels in the pathophysiology of obesity. Ion channels present diverse key functions, such as the maintenance of physiological homeostasis and cell proliferation. Cell biology and pharmacological experimental evidences demonstrate that proliferating cells exhibit ion channel expression, conductance, and electrical properties different from the resting cells. Thereby, a large variety of ion channels has been identified in the pathogenesis of obesity such as potassium, sodium, calcium and chloride channels, nicotinic acetylcholine receptor and transient receptor potential channels. The fundamental involvement of these channels on the generation of obesity leads to the progress in the knowledge about the mechanisms responsible for the obesity pathophysiology, consequently emerging as new targets for pharmacological modulation.

## Introduction

Obesity is an important disease associated to the excessive accumulation of body fat, leading to weight gain and the development of chronic disorders, including arterial hypertension, type 2 diabetes mellitus, dyslipidemias, sleep apnea, and cancer (Jeffreys et al., 2003; Van Gaal et al., 2006; Pischon et al., 2008; Whitlock et al., 2009; Vucenik and Stains, 2012; Singh et al., 2013). Currently, it affects 35% of adults aged 20+ with a rising prevalence (World Health Organization [WHO], 2014).

Multiple factors are involved in the origin of this disease, involving genetic determinants, as well as environmental, psychosocial and psychobiological factors (Comuzzie and Allison, 1998; Bocchieri et al., 2002; Noll et al., 2007). Meanwhile, regardless of its cause, the body weight results from a complex interaction between physio- and psychological components that control food intake and energy expenditure (Martinez, 2000).

Food intake is regulated centrally and it is influenced by the phenomena of hunger, satiety and appetite. Neurons located in several regions of the hypothalamus (ventromedial, lateral hypothalamic, arcuate and paraventricular nuclei) and in extra hypothalamic regions, including hindbrain (nucleus tractus solitarii), midbrain (ventral tegmental area) and forebrain (nucleus accumbens), are associated with the energetic homeostasis in human (Thaler et al., 2013).

Specifically, the arcuate nucleus (ARC) in the mediobasal hypothalamus contains different types of neurons correlated to the expression of orexigenic peptides, such as neuropeptide Y (NPY) and agouti-related peptide (AgRP), which stimulate food intake and reduce the body energy expenditure. In contrast, neurons expressing anorexigenic peptides derived from pro-opiomelanocortin (POMC), cocaine and amphetamine regulated transcript (CART) trigger opposite effects, such as decreased food intake and increased body energy expenditure (Bing et al., 1996; Schwartz et al., 2000; Broberger, 2005; Huda et al., 2006). In addition, the nucleus tractus solitarii (nTS) receives gustatory and visceroceptive signals, especially from vagal afferents synapse, and sends projections to different brain regions in order to regulate the food intake (Harding and Leek, 1973; Appleyard et al., 2005). Recently, several studies have suggest a role of ion channels in the regulation of synaptic function in the brain associated to the increase/decrease of expression of orexigen/anorexigen peptides and the energetic homeostasis (Olds and Xu, 2014).

The energetic balance has also multiple periphery influences, mainly the blood glucose levels, neurotransmitters, adipokines and cytokines, such as the hormones involved in glucose metabolism (adiponectin and resistin), inflammation [tumor necrosis factor-α (TNF-α) and interleukin 6 (IL6)], coagulation [plasminogen activator inhibitor type-l (PAI-1)], blood pressure (angiotensinogen and angiotensin II) and feeding behavior (leptin and ghrelin), which are responsible for the obesity progression (Chu et al., 2001; Yamauchi et al., 2001; Vendrell et al., 2004; Ran et al., 2006; Ohashi et al., 2014). Therefore, peripherally released hormones can regulate the development of obesity throughout the modulation of central events.

Leptin is a key adipokine in the regulation of energy intake/expenditure that shows concentrations proportional to body fat mass (Sinha and Caro, 1998). Leptin acts on the ARC stimulating the anorexigenic neurons and inhibiting the orexigenic ones (Druce and Bloom, 2006). Another important event promoted by this adipokine is the induction of post-translational processing of the precursor molecule of POMC, leading to the production of melanocortin peptides that present anorexigenic property (Broberger, 2005). Mutations affecting the leptin-melanocortin pathway involving genes encoding leptin and/or its receptor and the prohormone convertase enzyme that processes the POMC constitute the more present genetic alterations in obese (Farooqi and O’Rahilly, 2008). Ghrelin is produced in the stomach (Tschop et al., 2000; Wiedmer et al., 2007) and is the only peripheral hormone that stimulates the expression of orexigenic neuropeptides. Thus, it is known to induce increase in hunger and food intake (Huda et al., 2006). Before meals, the levels of ghrelin are raised, and after the ingestion of nutrients, its levels are suppressed (Tschop et al., 2001). Additionally, adiponectin is a hormone which is synthesized, exclusively, by adipocytes and acts inhibiting the hepatic glucose production, while increasing glucose uptake in muscle, as well as the oxidation of fatty acids in the liver and muscles, causing energy expenditure (Scherer et al., 1995; Kadowaki and Yamauchi, 2005). Therefore, obese individuals present low levels of adiponectin (Lindsay et al., 2002; Hajer et al., 2007).

The adipose tissue has the ability to store excess calories resulting in the process of hypertrophy and/or hyperplasia (Ferranti and Mozaffarian, 2008). The deposition and release of fatty acids from adipose tissue (as triglyceride) alters the food intake and energy expenditure. Moreover, different studies showed that adipose tissue is generally composed by 50% of adipocytes and the remaining 50% are cells such as preadipocytes, vascular cells, neural, leukocytes and cells of the immune system (Gustafson, 2010; Maenhaut and Van de Voorde, 2011). Based on functional and morphological differences, this tissue was divided into two main types, white adipose tissue (WAT) and brown adipose tissue (BAT). The WAT acts as an energy storage and is an active endocrine organ, releasing the free fatty acids and adipokines. Conversely, the BAT presents cells which are characterized by multilocular lipid droplets and an increased number of mitochondria, which express uncoupling protein 1 (UCP1), which uncouples the rates of substrate oxidation and ATP production by favoring a loss of protons and thus energy release. Thus, BAT is associated with thermogenesis (Tam et al., 2012).

Regarding the diagnosis of obesity, anthropometric criteria are the most often used, such as body mass index (BMI), measurement of waist circumference, waist-hip ratio, and skinfold test with the use of tweezers to evaluate the percentage of fat (Racette et al., 2003; McArdle et al., 2008). Additionally, there are other techniques for the assessment of body weight, including electrical bioimpedance (BIA), hydrostatic weighing and dual-energy X-ray absorptiometry (DEXA): BIA evaluates the resistance to a low frequency electrical current, considering that the current travels through the aqueous compartments and not through the greasy ones, where the flow impediment occurs (Kushner and Schoeller, 1986). The hydrostatic weighing determines the total volume of the body by analyzing the difference between the weight of an object in water and air, determining the density of the whole body (Schoeller et al., 1980). In DEXA analysis, the composition of soft body tissues is measured, by using the ratio of absorption of X-rays of different energies attached to adipose tissue mass (Kohrt, 1998).

Obesity treatment is based on multiple approaches that may encourage behavior change in long term, through energy-reduced diets, physical activity and/or exercise, pharmacotherapy and surgery (National Institutes of Health [NIH], 1991, 2000; Rosenbaum et al., 1997; Klein, 1999; Bray and Tartaglia, 2000). There is a consensus among the National Institutes of Health, the National Heart, Lung and Blood Institute and the North American Association for the Study of Obesity that the choice of treatment depends on the degree of obesity and the presence of comorbidities. Accordingly, for BMI ≥ 25 kg/m^2^, changes in lifestyle are recommended (physical activity, diet, and behavior), for BMI into 27.7–29.9 kg/m^2^, in the presence of comorbidities or for BMI ≥ 30 kg/m^2^, in the absence of comorbidities, the use of pharmacotherapy is recommended. The surgery for weight reduction is recommended for patients with severe obesity (BMI > 35 kg/m^2^) in the presence of comorbidities, or individuals with a BMI > 40 kg/m^2^ in the absence of comorbidities (National Institutes of Health [NIH], 2000).

Nowadays, the treatment of obesity remains a challenge. Most anti-obesity drugs currently prescribed target appetite regulation or decreasing in the absorption of nutrients (Bays, 2004). Most of them have significant side effects, and do not act specifically on regulatory functions of appetite in the hypothalamus, but exhibit a broad spectrum of actions. In many cases, beneficial effects remain limited (Heymsfield et al., 1999).

The most prescribed drugs for the treatment of obesity are sibutramine (serotonin and norepinephrine reuptake inhibitor) and orlistat (gastrointestinal lipase inhibitor), both prescribed for long-term use (Cannon and Kumar, 2009). The use of sibutramine for weight reduction has favorable effects on risk factors for cardiovascular disease (increasing high-density lipoprotein and decreasing levels of cholesterol and triglycerides in the blood; Dujovne et al., 2001). However, the use of this drug is contraindicated in patients with uncontrolled hypertension, coronary artery disease, cardiac arrhythmias, congestive heart failure or stroke, because it can increase blood pressure and heart rate (Kim et al., 2003). It is recommended the use of orlistat associated with multivitamin supplements, considering that orlistat affects the absorption of fat-soluble vitamins (A, D, E, and K) that are essential for the body (Cannon and Kumar, 2009). In obese patients, the benefits of this drug use is associated with lower incidence of type 2 diabetes, reduction of total cholesterol, LDL cholesterol and triglyceride levels as well as the levels of glycated hemoglobin (HbAlc) in diabetic (Aronne et al., 2009). Amfepramone (diethylcathinone) and fenproporex are also inhibitors of appetite, which act on the hypothalamic neurons, increasing the release of norepinephrine and stimulating the noradrenergic receptors, then, inhibiting hunger (Zaragoza et al., 2005; Cohen, 2009). Other medications have been evaluated in obese individuals, however, without formal indication for obesity treatment.

Because of ethical limitations on studying the susceptibility of human obesity, animal models of obesity has been proposed to investigate the pathophysiological changes caused by this disease (Pereira et al., 2003). Obesity has been induced in animals by nerve injury, endocrine disorders, genetic, and/or dietary modifications (Sclafani and Springer, 1976), which facilitates the study of the pathophysiology of obesity and allows advances in the development of new therapies.

Therefore, in view of the influence of obesity in the development of chronic diseases affecting the life expectation and the limitations of the current available treatment, this revision focused on the involvement of ion channels in the pathophysiology of obesity, as well as the role of these targets as new approaches toward the development of new anti-obesity drugs.

## Ion Channels In The Development Of Obesity

Ion channels play fundamental roles in diverse key functions, such as maintenance of physiological homeostasis, cell proliferation and signal transduction in a variety of cell types and different cell stages (Nilius and Droogmans, 2001; Chen et al., 2007). Cell biology and pharmacological experimental evidences demonstrate that proliferating cells exhibit ion channel expression, conductance, and electrical properties which are very different from the resting cells (Conti, 2007). In this context, the investigation of ion channels has been emerging as a new approach in the study of the pathogenesis of obesity.

### Ion Channels on Adipose Cell Proliferation

Obesity development results from the expansion of WAT and this process depends of stem cell proliferation (Gray and Vidal-Puig, 2007). This process is dependent of ion channels functionality (Chen et al., 2007). It is well-known that K^+^ channels are involved in cell proliferation, as the EGF-mediated mitogenic signal transduction process, required for voltage-gated K^+^ channels participating in G_1_/S-phase transition of the cell cycle (MacFarlane and Sontheimer, 2000). Previous studies reported the presence of voltage-gated K^+^ currents (K_V_) in BAT isolated from the interscapular fat pads of neonatal rats (Lucero and Pappone, 1989; Russ et al., 1993), WAT differentiated from rats (Ramirez-Ponce et al., 1996) and/or human preadipocytes (Ramirez-Ponce et al., 2003). Additionally, Bai et al. (2007) identified three types of K^+^ currents on human adipose tissue-derived stem cells (hASCs): a delayed rectifier-like K^+^ current (I_KDR_), a Ca^2+^-activated K^+^ current (I_KCa_) and a transient outward K^+^ current (I_to_). In addition, the mRNA of K_V_1.1, K_V_1.5, K_V_2.1, K_V_7.1, K_V_10.1, and K_V_11.1 has been shown to correspond to I_KDR_; mRNA of MaxiK (BK_Ca_), KCNN3 (SK_Ca_3) and KCNN4 (SK_Ca_4), corresponding to I_KCa_, and high mRNA levels of K_V_1.4, K_V_4.2, and K_V_4.3, contributing to I_to_. However, despite the expression of these different K^+^ channels in hASCs, only the *I*_KDR_ current has been associated to its proliferation (**Table 1**).

**Table 1:** Characterization of K^+^ channels associated to obesity.

Localization	Species	K^+^ channel subtype (gene)	Comments	Reference
Subcutaneous preadipocytes	Human	K_V_1.1 (*KCNA1*) K_V_1.5 (*KCNA5*) K_V_2.1 (*KCNB1*) K_V_7.1 (*KCNQ1*) K_V_10.1 (*KCNH1*) K_V_11.1 (*KCNH2*) K_Ca_1.1 (*KCNMA1*) K_V_4.2 (*KCND2*)	Promote preadipocytes proliferation	Bai et al., 2007; Hu et al., 2009
Brown adipocytes	Rat	K_V_ (?)	Associated with adipose cells proliferation	Pappone and Ortiz-Miranda, 1993
Central nervous system	Mice	K_ir_6.2 (*Kcnj11)*	Mediates the glucose-sensitive neuronal excitation and regulates food intake	Miki et al., 2001; Sohn, 2013
		K2P/K_V_3 [*Kcnk1–7, 9, 10, 12, 13, 15–17; Kcck18 (?)*]	Activated by high glucose levels and inhibits orexin/hypocretin release	Burdakov et al., 2006
		K_V_1.3 (*KCNA3)*	Regulation of energy homeostasis, body weight, and insulin resistance	Xu et al., 2003, 2004
	Rat	K_V_3.1 (*Kcnc1)*	Regulates response of taste cells to fat ingestion and the leaning to high fat intake	Gilbertson et al., 2005

Hu et al. (2009) showed the crucial participation of K^+^ channels in obesity. The knockdown or blockade with 4-aminopyridine and paxilline of K_Ca_1.1 and K_V_4.2 channels, respectively, related to I_to_, increased the cell number of human preadipocytes on G_0_/G_1_ phase, preventing cell cycle progression. Thus, these data indicate the importance of K^+^ channels activity to preadipocytes proliferation and differentiation, resulting in obesity installing. Additionally, in brown adipocytes culture obtained from rat, it has been demonstrated that the blockade of K_V_ channels with tetraethylammonium inhibits cell proliferation (Pappone and Ortiz-Miranda, 1993). The brown adipocytes are connected to metabolic energy expenditure and, therefore, are important to prevent fat accumulation. Thus, a balance in the activity of white and brown fat cells can be seen, regulated by K^+^ channels, that prevent fat deposition. It is still not clear the role of K^+^ channels in the genesis of obesity. The data presented here ponder a similar role of these channels on the proliferation of both white and brown preadipocytes. It becomes important, therefore, to determine which of these subtypes are predominant for the evolution of the cell cycle in those two cell types and how its dysfunction would lead to the development of obesity (**Table 1**).

Other ion channels have been associated with proliferation of preadipocytes. On 3T3-L1-preadipocytes, an establish a model for adipocyte differentiation, and visceral adipose tissue from both mice and human, Zhang et al. (2007) have detected the expression of TRPV1 and have shown that TRPV1 are important in preventing the proliferation and differentiation of preadipocytes. Accordingly, the treatment with capsaicin, a TRPV1 agonist, prevented the differentiation of these cells. Likewise, it has been evidenced the TRPV1 downregulation during adipogenesis of preadipocytes as well as on visceral adipose tissue from *db/db* and *ob/ob* mice, suggesting that TRPV1 function prevents adipogenesis and consequently obesity (Miller et al., 1996; Neal and Clipstone, 2002) (**Table 2**). Additionally, Hu et al. (2010) have detected Cl^-^ currents on human abdominal subcutaneous adipose tissue. Furthermore, it was verified the expression of chloride channel-3 (CIC-3) on this tissue and its blockade with tamoxifen reduced cell proliferation, suggesting the role of Cl^-^ channel in regulation of human preadipocyte proliferation.

**Table 2:** Characterization of TRPs associated to obesity.

Localization	Species	TRP channel (gene)	Comments	Reference
Adipocytes	Mice	TRPC1 *(Trpc1)* TRPC5 *(Trpc5)*	Channels related to the adiponectin production	Sukumar et al., 2012
		TRPM8 *(Trpm8)*	Menthol, a TRPM8 agonist, increases the expression of UCP1 and the level of p-PKA	Ma et al., 2012
3T3-LI- preadipocytes	Mice	TRPV1 *(Trpv1)*	Adipogenesis process is decreased	Zhang et al., 2007
BAT	Mice	TRPM8 *(Trpm8)*	Stimulation of this channel mediates BAT thermogenesis	Ma et al., 2012
		TRPA1 *(Trpa1)*	Cinnamaldehyde, a TRPA1 agonist, increases the UCP1 protein levels	Tamura et al., 2012
WAT	Mice	TRPV4 (*Trpv4)*	Regulator of oxidative metabolism, thermogenesis, and pro-inflammation gene	Ye et al., 2012
		TRPM2 (*Trpm2)*	Regulates of lipid metabolism	Zhang et al., 2012
Perivascular adipose tissue	Human	TRPC1 *(TRPC1)* TRPC5 *(TRPC5)*	Channels are related to the adiponectin production	Sukumar et al., 2012
Visceral and subcutaneous adipose tissues	Human	TRPV1 *(TRPV1)*	Adipogenesis process is decreased	Zhang et al., 2007
	Mice	TRPA1 *(Trpa1)*	Cinnamaldehyde, a TRPA1 agonist, reduces the visceral fat in both mice fed with a high-fat and high-sucrose	Tamura et al., 2012
TRCs	Mice	TRPM5 (*Trpm5)*	Disruption on the channel abolishes the sweet, umami, and bitter tastes	Zhang et al., 2003
EC cells	Rat	TRPA1 *(Trpa1)*	Allyl isothiocyanate and cinnamaldehyde, TRPA1 agonists, increase [Ca^2+^]_i_, 5-HT release and delay gastric emptying	Doihara et al., 2009; Nozawa et al., 2009
Whole body fat	Human	TRPV1 *(TRPV1)*	SNP Val585IIe is associated to weight loss	Hayes et al., 2000
	Mice	TRPM5 *(Trpm5)*	TRPM5^(-/-)^ mice present a loss on sucrose preference and gain less weight that WT mice	Glendinning et al., 2012
		TRPV1 *(Trpv1)*	TRPV1^(-/-)^ mice present equivalent energy intake that WT mice	Motter and Ahern, 2008

Therefore, all these data directs to a probable requirement for hyperpolarized state of preadipocytes, which can be achieved by the eﬄux of K^+^, inhibition of cation influx or even by Cl^-^ influx, to the progress of these cells in the cell cycle until reaching the needed maturity state for their proliferation and differentiation. Accordingly, drugs that prevent this progression by acting on ion channels involved could provide new tools for obesity therapy.

### Ion Channels on Central Food Intake Control

Some K^+^ channels have been shown to have an important role in the central regulation of food intake, energy expenditure, and glucose metabolism. The K_ir_6.2, suggested being the K_ATP_ pore-forming subunit (Karschin et al., 1998; Zawar et al., 1999; Miki et al., 2001), is expressed in neurons of ventromedial hypothalamic nucleus (VMH), POMC and melanin-concentrating hormone (MCH) neurons of lateral hypothalamic area (LHA) of mice and mediates neuronal glucose-excitation, acting as a glucose sensor during feeding. The defective K_ir_6.2 prevents ATP-mediated K_ATP_ blocking, leading to impaired glucose responsiveness by POMC neurons, resulting in glucose intolerance (Sohn, 2013). Additionally, this event has been observed in obese mice on a high-fat diet, resulting in loss of glucose sensitivity by these neurons, contributing to food intake overstimulation and obesity progress (Miki et al., 2001). These data corroborate Rowe et al. (1996), who showed that obese rats exhibit abnormal electrophysiological responses in hypothalamic glucose-sensors neurons to changes on extracellular glucose concentration, whereas lean rats respond normally. The role of K_ATP_ on regulating food intake behavior has been also shown to be important in preventing age-dependent obesity. It was shown that rapamycin-sensitive (mTOR) signaling is elevated in POMC neurons of old mice, causing silencing of these neurons and inhibition of leptin-induced release of the anorexigenic α-MSH, associated with upregulation of K_ATP_ channel activity and an aging-dependent high expression of K_ir_6.2 (Yang et al., 2012) (**Table 1**).

Leptin and insulin are well-recognized as anorexigenic hormones released from adipocytes and pancreatic beta cells, respectively (Williams et al., 2011b). Typically, leptin activates POMC neurons and leptin receptor (LepR)-expressing neurons of ventral premammilary nucleus (PMV), known to release of anorexigenic hormones (Cowley et al., 2001; Al-Qassab et al., 2009; Williams et al., 2010, 2011a). However, their effects on ion channels in the CNS are yet controversial. In the VMH, insulin receptors expressed by the SF-1 neurons activate K_ATP_ channels and suppress steroidogenic factor 1 (SF1) neuron activity, which resulted in diet-induced obesity (Klockener et al., 2011). In addition, insulin activates some arcuate NPY/AgRP neurons, which release orexigenic hormones, leading to hyperphagia state (Al-Qassab et al., 2009). Melanocortin is another hormone important in regulating body fat accumulation. Mutations on MC4R has been shown to induce obesity in rodents and humans (Huszar et al., 1997; Vaisse et al., 1998; Tallam et al., 2005), and its activation hyperpolarizes parasympathetic preganglionic neurons in brainstem via PKA-dependent activation of tolbutamide-sensitive K_ATP_ channels and depolarize the sympathetic ones, which could be one of the factors responsible to inducing obesity-associated hypertension. However, this data is more associated with an obesity consequence than a cause. The ion channels involved in melanocortin pathways to regulate food intake and obesity development have not yet been determined. Therefore, the effects of these hormones on ion channels activity in neurons that regulate food intake deserve an interesting focus for future insights attempting to better understand their relationship with obesity. Meanwhile, K_ATP_ channels appear to be promising targets to treatment of obesity, focusing in drugs acting in CNS neurons regulating food intake (**Table 1**).

K_V_1.3 has been implicated in the regulation of energy homeostasis, body weight and insulin resistance (Xu et al., 2003, 2004). This channel was shown to act as a metabolic sensor responding to insulin levels in mitral cells of olfactory bulb, and its removal increases the weight gain (Marks and Fadool, 2007; Biju et al., 2008). Mitral cells of the olfactory bulb function as internal chemical sensors of metabolic state by modulating K_V_ channels predominantly expressed in these neurons (Fadool et al., 2000). K_V_1.3 knockout mice fed a high-fat diet exhibit increased light-phase metabolism in addition to reduced weight gain, reduced levels of sugar, leptin and insulin in the blood, and increased energy expenditure (Xu et al., 2003, 2004; Li et al., 2006; Tucker et al., 2008; Fadool et al., 2011; Tucker K. et al., 2012; Tucker K.R. et al., 2012). Additionally, these knockout exhibited metabolic alterations including increase in energy expenditure and locomotor activity, resistance to diet- and genetic-induced obesity and increased insulin sensitivity (Fadool et al., 2004; Xu et al., 2004; Tucker et al., 2008). Therefore, linking to the role of K_ATP_ channels in hypothalamic areas to regulate food instinct, the K_V_1.3 channels seem to have an important role at the CNS too. These channels appear to be important in regulating food drive through the olfactory effects which are responsible for stimulating food intake (**Table 1**).

Nicotinic receptors have been shown to have an important role to prevent fat accumulation and to induce fat degradation. In a model of type 2 diabetes, the homozygous leptin-resistant *db*/*db* obese mouse, Marrero et al. (2010) measured the effects of a novel α7 nAChR-selective agonist, TC-7020, and showed reduction of food intake and weight gain. These parameters were reversed by using a janus kinase 2 (JAK2) specific inhibitor (AG-290), demonstrating that the α7 nAChRs plays an important role in the body weight control and it involves JAK2 signal transducer and activator of transcription 3 (STAT3) signaling pathways. Similarly, C57BL/6J mice with a high-fat diet treated with galantamine, an acetylcholinesterase (AChE) inhibitor that enhances cholinergic signaling and also acts as a positive allosteric modulator of a7 nAChR, presented reduction on food intake, body weight and abdominal adiposity as well as an improvement on blood glucose, insulin resistance, and hepatic steatosis (Satapathy et al., 2011) (**Table 3**).

**Table 3:** Characterization of nAChRs associated to obesity.

Localization	Species	nAChRs (gene)	Comments	Reference
Central nervous system	Human	α7 (*CHRNA7)* α/β (*CHRNA1, 2, 3, 4, 5, 6, 9, 10 and CHRNB1, 2, 3, 4)*	Associated to the energetic metabolism regulation	Jo et al., 2002
	Rat	α3 (*Chrna3)* β4 (*Chrnb4)*	Distributed in both the peripheral and central nervous system. Associated to food intake regulation	Dwyer et al., 2008
	Mice	α4,7 (*Chrna4, 7)* β2 (*Chrnb2)*	Express in hypothalamus regulating appetite, food consumption, and body mass	Jo and Role, 2002
		α3 (*Chrna3)* β4 (*Chrnb4)*	Regulates food intake	Mineur et al., 2011
Adipose tissue	Human	α7 (*CHRNA7)*	Gene expression is reduced in obese individuals	Cancello et al., 2012
		α2 (*CHRNA2)*	Gene expression is associated to overweight/obesity	Kim, 2008
		α3–5 (*CHRNA3,4,5)*	Associated to alteration on BMI	Freathy et al., 2011; Zhu et al., 2013
		α3, 5 (*CHRNA3,5)* β3, 4 (*CHRNB3,4)*	Associated to abdominal obesity	Zhu et al., 2014
	Rat	α1–7, 9, 10 (*Chrna 1, 2, 3, 4, 5, 6, 7, 9, 10)* β1–4 (*Chrnb1, 2, 3,4)* δ (*Chrnd)* ε (*Chrne)*	Promote nicotine-induced adipocytes adiponectin and FFA release	Liu et al., 2004
	Mice	α2 (*Chrna2)* β1–2 (*Chrnb1,2)*	Alterations in lean body mass and fat storage	Somm et al., 2014

Cigarette smoking habits have been associated with less body fat distribution, body weight, insulin resistance and obesity (Chiolero et al., 2008; Clair et al., 2011). Indeed, the smoking cessation increases body weight (Perkins, 1993). Experimentally, nicotine administration to rodents and humans suppresses appetite, increases energy expenditure and alters feeding behavior (Jo et al., 2002; Fornari et al., 2007; Zoli and Picciotto, 2012). The mechanisms involved in nicotine-induced decrease in body weight have been correlated to both central and peripheral nicotinic cholinergic signaling. In CNS, nAChRs composed by both α7 and α/β subunits were detected throughout areas of the hypothalamus that regulate appetite, food consumption and body mass. In previous studies, it was revealed from moderate to high levels of α4, α7, and β2-mRNAs in hypothalamus, with particularly prominent expression in the supraoptic or suprachiasmatic nuclei and lateral hypothalamus (Jo and Role, 2002). Moreover, although the α3β4 nAChR is the predominant nicotinic receptor in the peripheral nervous system, it is less widely distributed in the rat brain (Dwyer et al., 2008) and has been related to nicotine effects on mice hypothalamus to decrease food intake. In particular, the activation of α3β4 nAChRs in POMC cells in the ARC decreases food intake and increases energy expenditure (Williams and Schwartz, 2005; Mineur et al., 2011) (**Table 3**).

The gamma-aminobutyric acid type A receptor (GABA_A_) has been associated to the body weight regulation. Tong et al. (2008) showed that inhibition of GABA release from AgRP neurons of mice prevents obesity. Additionally, Vong et al. (2011) showed a lean phenotype in the presence of defective synaptic release of GABA from NPY/AgRP neurons. This effect was associated to a decrease on inhibitory post-synaptic currents (IPSCs) in anorexigenic POMC neurons. Beyond this, Wu et al. (2009) have showed that inhibition of GABA_A_ receptor with the antagonist bicuculline, in the parabrachial nucleus (PBN), a region that regulates taste reactivity (Higgs and Cooper, 1996), decreases the feeding and increases the loss of body weight. Thus, there are evidences linking the inhibitory effect of GABA_A_ receptors in anorexigenic hypothalamic neurons that may be responsible for promoting obesity development.

Monosodium glutamate has been shown to cause hypothalamic damages (Kaufhold et al., 2002) and the chronic overconsumption of the aminoacid glutamate (GLU) promotes obesity (Hermanussen and Tresguerres, 2003). In this view, the selective GLU-gated calcium channels antagonist, memantine, has been reported to display neuroprotective effects. Additionally, obese young women since childhood or after the first pregnancy treated with memantine presented protection on physiological regulation of appetite affected by high nutritional GLU, as well as being led to marked body weight reduction within a few days (Hermanussen and Tresguerres, 2005). Probably activation of Ca^2+^ channels in the hypothalamus, such as that produced by glutamate on its ionotropic NMDA-R, promotes release of orexigenic hormones, affecting the nutritional status and triggering obesity. However, more works in this field should be conducted to better characterize the role of these ion channels.

### Ion Channels on Sleep-Vigilant Cycle Dysfunction-induced Obesity

There are few studies linking sleep and metabolism, being this interplay only currently receiving attention. In a study made by Uebele et al. (2009) using mice with deletion of Ca_V_3.1 channels (*Cacna1g* KO mice), a model that promotes fragmented sleep, the potential link between sleep and obesity was investigated. The authors found that KO mice presented less weight gain and amount of fat after inducing them to a high-fat diet (HFD), compared to the wild type mice (WT). Moreover, it was showed that the resistance on weight gain observed to KO mice was due to metabolic rate changes in these animals, in a manner that did not affect core body temperature. Furthermore, the authors assessed a possible similar effect using a T-type Ca^2+^ channel antagonist (TTA-A_2_) and, initially, they observed that the inhibition of T-type Ca^2+^ of WT mice caused sedation and decreased on active wake, consistent with altering thalamocortical neuronal activity. Additionally, these WT mice presented less weight gain after inhibition of T-type Ca^2+^ and, interestingly, this difference was observed only when the mice received HFD. All the results were similar to that observed on *Cacna1g* KO mice. Additionally, animals fed with HFD presented reduced food intake, not on the active phase, but in the inactive phase. These effects probably result from a better alignment of diurnal feeding patterns with daily changes in circadian physiology and, potentially, an increased metabolic rate during the active phase. Thus, these data suggest a role for Ca_V_3.1 in co-regulating sleep and weight maintenance and data from pharmacological studies demonstrate that potent and selective T-type calcium channel antagonists reduce– wakefulness, diet-induced weight gain, and improve- body composition, suggesting this ion channel class may provide a novel therapeutic target for the treatment of obesity.

Additionally, it has been shown that elevated glucose levels, reached, e.g., after meal, activate a two-pore domain K^+^ channel (K2P) and inhibits orexin/hypocretin release by LHA neurons, regulating the sleep-vigilant cycle (Burdakov et al., 2006). Thus, impaired K2P function can emerge as a reason of hyperphagia promoted by release of high amounts of this hormone associated with sleep-vigilant cycle dysfunction (**Table 1**).

Therefore, there must be an interplay between Ca^2+^ and K^+^ channels that regulates sleep-vigilant cycle and food intake as well, emerging as interesting targets in the CNS to treat obesity, especially the sleeplessness.

### Ion Channels on Peripheral Food Intake Control

Free fatty acids are able to activate peripherally taste receptor cells (TRCs; Fukuwatari et al., 2003), the stimulus involves an interaction between the tastant and ion channels or receptors localized in most cases on the apical membranes of the TRCs. The initial events in the taste transduction of free fatty acids include an inhibition of delayed rectifying potassium channels (DRK) associated with K_V_3.1 channels (Gilbertson et al., 1997, 1998, 2005). Gilbertson et al. (2005) have demonstrated that DRK currents are inhibited in obese-resistant rats (O-R), but not in obesity-prone (O-B) test cells, in response to polyunsaturated fat acids (PUFAs), and this effect was associated with the greater activation of taste cells in O-R than that in O-B. Additionally, TRCs in O-R would be activated to a greater degree by PUFAs than those from O-B. Likewise, the DRK channels were not found in O-R, indicating that PUFAs do not inhibit K^+^ currents by these channels in O-B, and they are the most responsible for the taste cells inhibition in obese rats, which might explain the preference of these obese rats to a high fat dietary, while the resistant-rats tend to eat much less (**Table 1**).

TRPM5 is another ion channel abundantly identified in TRCs which participates in sweet, umami, and bitter perception. The disruption of this channel gene in mice abolishes the transduction of these tastes (Pérez et al., 2002; Zhang et al., 2003). TRPM5^(-/-)^ mice were shown to present the same pattern of food intake and gained less weight than wide-type mice when fed a high-sugar diet, being this effect probably due to the loss of sucrose preference (Glendinning et al., 2012). Interestingly, these data corroborate Gilbertson et al. (2005), as showed previously, that demonstrated that inhibited state of taste cells leads to obesity sensitivity. Additionally, TRPM5^(-/-)^ mice also abolished the preference for fat and reduced the fatty acid-induced [Ca^2+^]_c_ increase, suggesting that TRPM5 also plays a key role in lipid taste perception (Oike et al., 2006; Sclafani et al., 2007; Liu et al., 2011) (**Table 2**).

### Ion Channels on Body Energetic Expenditure

Some ion channels have been associated with the regulation of energetic expenditure. The nicotinic cholinergic signaling, in special, in metabolic tissue, is one of them. Liu et al. (2004) have shown that α1–7, 9–10, β1–4, δ and ε subunit mRNAs are expressed in rat adipocytes, and the release of FFA by this cell has been known to increase nicotine concentration. Recent studies of Somm et al. (2014) have shown that in mouse WAT, α2 and β1 were the most expressed nAChR subunits. Meantime, mouse BAT expresses α2 and comparable amounts of β1 and β2 nAChR subunits. Then, although in a α7β2 nAChR^(-/-)^ mice model it was verified the unaltered body weight, a decreased fat storage was observed. Additionally, Cancello et al. (2012) have demonstrated the expression of α7 nAChR in isolated mature adipocytes and human subcutaneous adipose tissue (SAT). Meanwhile, the expression of this receptor was associated with decreased SAT obtained from obese human compared to normal-weight human. In addition, α7 nAChR had 75% lower expression in mature adipocytes isolated from morbidly obese human compared to adipocytes from normal-weight human. These receptors modulates inflammatory gene expression in human adipocytes and are linked to adipocyte lipid catabolism (**Table 3**).

In human studies, polymorphisms in genes encoding nAChRs have been focused to examine the potential involvement of nicotinic receptors variants on the distribution of body fat. The SNP in the nAChR α2 subunit gene (CHRNA2) was associated with overweight/obesity (Kim, 2008). There was no association between the BMI and the genotype of 15q25 SNP (CHRNA5-CHRNA3-CHRNB4 gene region) in the never smokers, however, in smoking individuals it was observed an association between the 15q25 variant and BMI, highlighting the smoking habit as a probable cause of reduced BMI and obesity resistance (Freathy et al., 2011; Zhu et al., 2013) (**Table 3**).

Regarding TRP channels, genetic approaches of human TRPV1 showed a SNP, Val585Ile, associated with weight loss. It was showed that the presence of Val/Val and Val/IIe variants leads to the loss of twice as much abdominal fat in response to vanilloids, as those with IIe/IIe (Hayes et al., 2000; Snitker et al., 2009). Indeed, the energy intake is decreased in humans that consume red chili pepper (Yoshioka et al., 1999) and the repeated intake of CH-19 Sweet, a non-pungent red pepper, reduces the body weight and suppresses body fat accumulation due to the sympathetic nervous activation, which leads to fat acids mobilization (Kawabata et al., 2006). The balance of the literature suggests that both capsaicin and capsiate augment energy expenditure and enhance fat oxidation, especially at high doses (Ludy et al., 2011). In addition, Ma et al. (2012) showed a functional TRPM8 expression on BAT, associated with metabolic energy expenditure and the use of menthol, a TRPM8 activator, increases the expression of UCP1, located in the inner mitochondrial membrane, responsible for thermogenesis in BAT (Vogler et al., 2008). TRPM2 has also been shown to be involved in the energy expenditure as evidenced for Zhang et al. (2012), but conversely, TRPM2^(-/-)^ mice were resistant to obesity after 4–10 months of high-fat feeding. These animals presented greater energy expenditure and elevated expression of lipid metabolic genes in WAT. Additionally, white adipocytes from TRPV4^(-/-)^ mice fed a high-fat diet were smaller and exhibited enhancement in the energy expenditure, due to the elevated levels of UCP1 expression. Thus, Ye et al. (2012) suggest that TRPV4 negatively regulates oxidative metabolism. In addition, Kusudo et al. (2012) have shown that inactivation of the TRPV4 gene induces an increase in TRPC3 and TRPC6 expression and calcineurin activity in mice. The energy metabolism was altered due to the expression of genes involved in fuel oxidative in skeletal muscle, contributing to the resistance to obesity. Likewise, Tamura et al. (2012) have reported that the supplementation with cinnamaldehyde, a TRPA1 agonist, increased the UCP1 protein levels in the interscapular BAT and reduced the visceral fat in mice fed with diet rich in fat or sucrose. Therefore, these data support the key role of TRPs on regulating energy expenditure involving specially the activity of BAT (**Table 2**).

It has also been extensively reported that intracellular Ca^2+^ ([Ca^2+^]_i_) appears to be involved in metabolic derangements, including obesity and insulin resistance. It was previously reported that agouti-induced obese mice present high basal [Ca^2+^]_i_ as well as increased Ca^2+^ influx rate, also associated with high expression and activity of fatty acid synthase (FAS) and increased triglycerides accumulation in 3T3-L1 adipocytes (Zemel et al., 1995). Interestingly, FAS activity has been prevented by Ca^2+^ channel blockade with nitrendipine (Jones et al., 1996). Moreover, it was reported that chronic hyperinsulinemia induces an increase in FAS mRNA level and activity in both rat liver and WAT (Chakrabarty and Leveille, 1969). In this context, Kim et al. (1996) reported that treatment of obese mice with nifedipine normalized both FAS mRNA and plasma insulin levels, associated with a reduction of fat pad mass in agouti-induced obese mice, together with an increase in the skeletal muscle weigh increase, which can be associated with the use of energy from adipose store by muscle cells. Indeed, it was also observed that the treatment corrected the reduction in core temperature of obese mice, which can be explained by the increase in energy expenditure by skeletal muscle. This effect was associated with a possible reduction on Ca^2+^ levels on pancreatic β-cells, reducing the insulin secretion, or due to an improvement in insulin sensitivity on these mice. Alternatively, it is not discarded that Ca^2+^ can acts in transcriptional level to increase the FAS expression. Therefore, nifedipine was able to reduce lipogenesis and insulin resistance in obese mice. Early evidence has already been reported, according to which the anti-obese effect of a Ca^2+^ channel blocker, benidipine, which reduced mice body weight associated with the increase in blood flow to BAT, functions as the main thermogenic organ (Yoshida et al., 1994).

Together, these data bring interesting information regarding nicotinic, TRPs and calcium channels as key ion channels responsible for regulating energy expenditure. Therefore, new drugs targeting these channels could emerge as new approaches in the field of obesity treatment.

### Ion Channels on Accelerated Gastric Emptying Leading to Overeating

The accelerated gastric emptying and thus reduced nutrient absorption has been linked with food overconsumption and obesity development. Nozawa et al. (2009) have showed the expression of TRPA1 in the gastrointestinal tissues and enterochromaffin cells (ECs) in humans, mice and rats. The stimulation of TRPA1 by allyl isothiocyanate (AITC) and cinnamaldehyde increased 5-hydroxytryptamine (5-HT) release from EC cells, which is known to accelerate the gastric emptying in human and it is known to the overconsumption on a HFD and subsequent obesity (Wright et al., 1983; Zahorska-Markiewicz et al., 1986; Tosetti et al., 1996) (**Table 2**). Furthermore, there are reports suggesting that gastric emptying of solid food is accelerated in obese subjects, which precipitates hunger and frequent eating (Wright et al., 1983; Tosetti et al., 1996). In obese humans, accelerated gastric emptying may be one of the contributing factors to overconsumption on a HFD and subsequent obesity (Wright et al., 1983; Zahorska-Markiewicz et al., 1986; Tosetti et al., 1996). In this context, Li et al. (2013) reported Ca^2+^ and K^+^ currents in antral circular smooth muscle cell. The diet-induced obese-prone rats exhibit accelerated gastric emptying attributed to a higher density of L-type Ca^2+^ currents (I_Ba,L_) and inactivation or low density of K^+^ currents, being them the responsible for the high Ca^2+^ currents amplitude in these cells and thus increasing gastric emptying, leading to overconsumption and obesity.

## Perspectives

In view of the large amount of information regarding the involvement of ion channels in the obesity development, as well as the development of suitable animal models, a new unexplored aspect arises to establish novel alternatives for obesity treatment having as targets deregulated molecular aspects in the genesis of this disease. Various ion channels have been identified in several cell types as important regulators of some functions related to the development of obesity. The K^+^ channels were the most well-studied and have been associated with the proliferation of adipose cells, central and peripheral regulation of food intake and gastric emptying time, all preponderant for the development of obesity. Other channels, such as TRPs and nicotinic receptors, are involved in many of these functions. The Ca^2+^ channels, although still not better explored, has been associated particularly in unusual obesity factors, such as disruption of the sleep-wake cycle, as well as chloride channels not yet explored, but identified in some cell types involved in obesity. In this sense, further research targeting important ion channels in the pathogenesis of obesity are promising for the development of more effective drugs to treat the disease. Accordingly, drugs that act on the ion channels described, specially K^+^ channels, may be promising to the further treatment of obesity.

## Author Contributions

LV, IS, and LP made the major part of research, designed and write the manuscript; BdS guided the preparation of the work.

## Conflict of Interest Statement

The authors declare that the research was conducted in the absence of any commercial or financial relationships that could be construed as a potential conflict of interest.

## References

[B1] Al-Qassab H. Smith M. A. Irvine E. E. Guillermet-Guibert J. Claret M. Choudhury A. I. (2009). Dominant role of the p110 beta isoform of PI3K over p110 alpha in energy homeostasis regulation by POMC and AgRP neurons. *Cell Metab.* 10 343–354. 10.1016/j.cmet.2009.09.008 19883613 PMC2806524

[B2] Appleyard S. M. Bailey T. W. Doyle M. W. Jin Y. H. Smart J. L. Low M. J. (2005). Proopiomelanocortin neurons in nucleus tractus solitarius are activated by visceral afferents: regulation by cholecystokinin and opioids. *J. Neurosci.* 25 3578–3585. 10.1523/JNEUROSCI.4177-04.2005 15814788 PMC6725389

[B3] Aronne L. J. Nelinson D. S. Lillo J. L. (2009). Obesity as a disease state: a new paradigm for diagnosis and treatment. *Clin. Cornerstone* 9 9–29. 10.1016/S1098-3597(09)80002-1 19789061

[B4] Bai X. Ma J. Pan Z. Song Y. H. Freyberg S. Yan Y. (2007). Electrophysiological properties of human adipose tissue-derived stem cells. *Am. J. Physiol. Cell Physiol.* 293 C1539-C1550. 10.1152/ajpcell.00089.2007 17687001

[B5] Bays H. E. (2004). Current and investigational antiobesity agents and obesity therapeutic treatment targets. *Obes. Res.* 12 1197–1211. 10.1038/oby.2004.151 15340100

[B6] Biju K. C. Marks D. R. Mast T. G. Fadool D. A. (2008). Deletion of voltage-gated channel affects glomerular refinement and odor receptor expression in the olfactory system. *J. Comp. Neurol.* 506 161–179. 10.1002/cne.21540 18022950 PMC2650277

[B7] Bing C. Wang W. Pickavance L. Williams G. (1996). The central regulation of energy homeostasis: roles of neuropeptide Y and other brain peptides. *Biochem. Soc. Trans.* 24 559–565. 10.1042/bst0240559 8736803

[B8] Bocchieri L. E. Meana M. Fisher B. L. (2002). A review of psychosocial outcomes for surgery for morbid obesity. *J. Psychosom. Res.* 52 155–165. 10.1016/S0022-3999(01)00241-0 11897234

[B9] Bray G. A. Tartaglia L. A. (2000). Medicinal strategies in the treatment of obesity. *Nature* 404 672–677. 10.1038/35007544 10766254

[B10] Broberger C. (2005). Brain regulation of food intake and appetite: molecules and networks. *J. Intern. Med.* 258 301–327. 10.1111/j.1365-2796.2005.01553.x 16164570

[B11] Burdakov D. Jensen L. T. Alexopoulos H. Williams R. H. Fearon I. M. O’Kelly I. (2006). Tandem-pore K+ channels mediate inhibition of orexin neurons by glucose. *Neuron* 50 711–722. 10.1016/j.neuron.2006.04.032 16731510

[B12] Cancello R. Zulian A. Maestrini S. Mencarelli M. Della Barba A. Invitti C. (2012). The nicotinic acetylcholine receptor α7 in subcutaneous mature adipocytes: downregulation in human obesity and modulation by diet-induced weight loss. *Int. J. Obes. (Lond.)* 36 1552–1557. 10.1038/ijo.2011.275 22270376

[B13] Cannon C. P. Kumar A. (2009). Treatment of overweight and obesity: lifestyle, pharmacological, and surgical options. Obesity as a disease state. *Clin. Cornerstone* 9 55–71. 10.1016/S1098-3597(09)80005-7 19789064

[B14] Chakrabarty K. Leveille C. A. (1969). Acetyl CoA carboxylase and fatty acid synthetase activities in liver and adipose tissue of meal-fed rats. *Proc. Soc. Exp. Biol. Med.* 131 1051–1054. 10.3181/00379727-131-34038 5791775

[B15] Chen L. X. Zhu L. Y. Jacob T. J. Wang L. W. (2007). Roles of volume-activated Cl- currents and regulatory volume decrease in the cell cycle and proliferation in nasopharyngeal carcinoma cells. *Cell Prolif.* 40 253–267. 10.1111/j.1365-2184.2007.00432.x 17472731 PMC6496325

[B16] Chiolero A. Faeh D. Paccaud F. Cornuz J. (2008). Consequences of smoking for body weight, body fat distribution, and insulin resistance. *Am. J. Clin. Nutr.* 87 801–809. 18400700 10.1093/ajcn/87.4.801

[B17] Chu N. F. Spiegelman D. Hotamisligil G. S. Rifai N. Stampfer M. Rimm E. B. (2001). Plasma insulin, leptin, and soluble TNF receptors levels in relation to obesity-related atherogenic and thrombogenic cardiovascular disease risk factors among men. *Atherosclerosis* 157 495–503. 10.1016/S0021-9150(00)00755-3 11472752

[B18] Clair C. Chiolero A. Faeh D. Cornuz J. Marques-Vidal P. Paccaud F. (2011). Dose-dependent positive association between cigarette smoking, abdominal obesity and body fat: cross-sectional data from a population-based survey. *BMC Public Health* 11:23 10.1186/1471-2458-11-23 PMC3025841 21223575

[B19] Cohen P. A. (2009). Imported fenproporex-based diet pills from Brazil: a report of two cases. *J. Gen. Intern. Med.* 24 430–433. 10.1007/s11606-008-0878-4 19096898 PMC2642570

[B20] Comuzzie A. G. Allison B. B. (1998). The search for human obesity genes. *Science* 280 1374-1377. 10.1126/science.280.5368.1374 9603720

[B21] Conti M. (2007). Targeting ion channels for new strategies in cancer diagnosis and therapy. *Curr. Clin. Pharmacol.* 2 135–144. 10.2174/157488407780598153 18690861

[B22] Cowley M. A. Smart J. L. Rubinstein M. Cerdán M. G. Diano S. Horvarth T. L. (2001). Leptin activates anorexigenic POMC neurons through a neural network in the arcuate nucleus. *Nature* 411 480–484. 10.1038/35078085 11373681

[B23] Doihara H. Nozawa K. Kawabata-Shoda E. Kojima R. Yokoyama T. Ito H. (2009). TRPA1 agonists delay gastric emptying in rats through serotonergic pathways. *Naunyn Schmiedebergs Arch. Pharmacol.* 380 353–357. 10.1007/s00210-009-0435-7 19629446

[B24] Druce M. Bloom S. R. (2006). The regulation of appetite. *Arch. Dis. Child.* 91 183–187. 10.1136/adc.2005.073759 16428368 PMC2082685

[B25] Dujovne C. A. Zavoral J. H. Rowe E. Mendel C. M. (2001). Effects of sibutramine on body weight and serum lipids: a double-blind, randomized, placebo-controlled study in 322 overweight and obese patients with dyslipidemia. *Am. Heart J.* 142 489–497. 10.1067/mhj.2001.117510 11526363

[B26] Dwyer J. B. Broide R. S. Leslie F. M. (2008). Nicotine and brain development. *Birth Defects Res. C Embryo Today* 84 30–44. 10.1002/bdrc.20118 18383130

[B27] Fadool D. A. Tucker K. Pedarzani P. (2011). Mitral cells of the olfactory bulb perform metabolic sensing and are disrupted by obesity at the level of the Kv1.3 ion channel. *PLoS ONE* 6:24921 10.1371/journal.pone.0024921 PMC3178571 21966386

[B28] Fadool D. A. Tucker K. Perkins R. Fasciani G. Thompson R. N. Parsons A. D. (2004). Kv1.3 channel gene-targeted deletion produces “Super-Smeller Mice” with altered glomeruli, interacting scaffolding proteins, and biophysics. *Neuron* 41 389–404. 10.1016/S0896-6273(03)00844-4 14766178 PMC2737549

[B29] Fadool D. A. Tucker K. Phillips J. J. Simmen J. A. (2000). Brain insulin receptor causes activity-dependent current suppression in the olfactory bulb through multiple phosphorylation of Kv1.3. *J. Neurophysiol.* 83 2332–2348. 10758137 10.1152/jn.2000.83.4.2332 PMC4326263

[B30] Farooqi I. S. O’Rahilly S. (2008). Mutations in ligands and receptors of the leptinmelanocortin pathway that lead to obesity. *Nat. Clin. Pract. Endocrinol. Metab.* 4 569–577. 10.1038/ncpendmet0966 18779842

[B31] Ferranti S. Mozaffarian D. (2008). The perfect storm: obesity, adipocyte dysfunction, and metabolic consequences. *Clin. Chem.* 54 945–955. 10.1373/clinchem.2007.100156 18436717

[B32] Fornari A. Pedrazzi P. Lippi G. Picciotto M. R. Zoli M. Zini I. (2007). Nicotine withdrawal increases body weight, neuropeptide Y and Agouti-related protein expression in the hypothalamus and decreases uncoupling protein-3 expression in the brown adipose tissue in high-fat fed mice. *Neurosci. Lett.* 411 72–76. 10.1016/j.neulet.2006.10.014 17052838

[B33] Freathy R. M. Kazeem G. R. Morris R. W. Johnson P. C. Paternoster L. Ebrahim S. (2011). Genetic variation at CHRNA5-CHRNA3-CHRNB4 interacts with smoking status to influence body mass index. *Int. J. Epidemiol.* 40 1617–1628. 10.1093/ije/dyr077 21593077 PMC3235017

[B34] Fukuwatari T. Shibata K. Iguchi K. Saeki T. Iwata A. Tani K. (2003). Role of gustation in the recognition of oleate and triolein in anosmic rats. *Physiol. Behav.* 78 579–583. 10.1016/S0031-9384(03)00037-4 12782211

[B35] Gilbertson T. A. Fontenot D. T. Liu L. Zhang H. Monroe W. T. (1997). Fatty acid modulation of K+ channels in taste receptor cells: gustatory cues for dietary fat. *Am. J. Physiol.* 272 C1203-C1210. 10.1152/ajpcell.1997.272.4.C1203 9142845

[B36] Gilbertson T. A. Liu L. Kim I. Burks C. A. Hansen D. R. (2005). Fatty acid responses in taste cells from obesity-prone and -resistant rats. *Physiol. Behav.* 86 681–690. 10.1016/j.physbeh.2005.08.057 16249010

[B37] Gilbertson T. A. Liu L. York D. A. Bray G. A. (1998). Dietary fat preferences are inversely correlated with peripheral gustatory fatty acid sensitivity. *Ann. N. Y. Acad. Sci.* 855 165–168. 10.1111/j.1749-6632.1998.tb10560.x 9929599

[B38] Glendinning J. I. Gillman J. Zamer H. Margolskee R. F. Sclafani A. (2012). The role of T1r3 and Trpm5 in carbohydrate-induced obesity in mice. *Physiol. Behav.* 107 50–58. 10.1016/j.physbeh.2012.05.023 22683548 PMC3409339

[B39] Gray S. L. Vidal-Puig A. J. (2007). Adipose tissue expandability in the maintenance of metabolic homeostasis. *Nutr. Rev.* 65 S7–S12. 10.1111/j.1753-4887.2007.tb00331.x 17605308

[B40] Gustafson B. (2010). Adipose tissue, inflammation and atherosclerosis. *J. Atheroscler. Thromb.* 17 332–341. 10.5551/jat.3939 20124732

[B41] Hajer G. R. Van Der Graaf Y. Olijhoek J. K. Edlinger M. Visseren F. L. (2007). Low plasma levels of adiponectin are associated with low risk for future cardiovascular events in patients with clinical evident vascular disease. *Am. Heart J.* 154 750.e1–750.e7. 10.1016/j.ahj.2007.07.013 17893004

[B42] Harding R. Leek B. F. (1973). Central projections of gastric afferent vagal inputs. *J. Physiol.* 228 73–90. 10.1113/jphysiol.1973.sp010073 4686026 PMC1331227

[B43] Hayes P. Meadows H. J. Gunthorpe M. J. Harries M. H. Duckworth D. M. Cairns W. (2000). Cloning and functional expression of a human orthologue of rat vanilloid receptor-1. *Pain* 88 205–215. 10.1016/S0304-3959(00)00353-5 11050376

[B44] Hermanussen M. Tresguerres J. A. F. (2003). Does high glutamate intake cause obesity? *J. Pediatr. Endocrinol. Metab.* 16 965–968. 10.1515/JPEM.2003.16.7.965 14513871

[B45] Hermanussen M. Tresguerres J. A. F. (2005). A new anti-obesity drug treatment: first clinical evidence that, antagonizing glutamate-gated Ca2+ ion channels with memantine normalises binge-eating disorders. *Econ. Hum. Biol.* 3 329–337. 10.1016/j.ehb.2005.04.001 15886075

[B46] Heymsfield S. B. Greenberg A. S. Fujioka K. Dixon R. M. Kushner R. Hunt T. (1999). Recombinant leptin for weight loss in obese and lean adults: a randomized, controlled, dose-escalation trial. *JAMA* 282 1568–1575. 10.1001/jama.282.16.1568 10546697

[B47] Higgs S. Cooper S. J. (1996). Hyperphagia induced by direct administration of midazolam into the parabrachial nucleus of the rat. *Eur. J. Pharmacol.* 313 1–9. 10.1016/0014-2999(96)00446-3 8905322

[B48] Hu H. He M. L. Tao R. Sun H. Y. Hu R. Zang W. J. (2009). Characterization of ion channels in human preadipocytes. *J. Cell. Physiol.* 218 427–435. 10.1002/jcp.21617 18942098

[B49] Hu H. Li D. L. Fan L. Ren J. Wang S. P. Jia B. (2010). Involvement of volume-sensitive Cl- channels in the proliferation of human subcutaneous pre-adipocytes. *Clin. Exp. Pharmacol. Physiol.* 37 29–34. 10.1111/j.1440-1681.2009.05223.x 19515064

[B50] Huda M. S. B. Wilding J. P. H. Pinkney J. H. (2006). Appetite regulatory peptides. Gut peptides and the regulation of appetite. *Obes. Rev.* 7 163–182. 10.1111/j.1467-789X.2006.00245.x 16629873

[B51] Huszar D. Lynch C. A. Fairchild-Huntress V. Dunmore J. H. Fang Q. Berkemeier L. R. (1997). Targeted disruption of the melanocortin-4 receptor results in obesity in mice. *Cell* 88 131–141. 10.1016/S0092-8674(00)81865-6 9019399

[B52] Jeffreys M. McCarron P. Gunnell D. McEwen J. Smith G. D. (2003). Body mass index in early and mid-adulthood, and subsequent mortality: a historical cohort study. *Int. J. Obes. Relat. Metab. Disord.* 27 1391–1397. 10.1038/sj.ijo.0802414 14574351

[B53] Jo Y. H. Role L. W. (2002). Differential modulation of synaptic transmission by nicotinic and muscarinic-receptor mediated pathways in lateral hypothalamus. *J. Neurophysiol.* 22 4794–4804.

[B54] Jo Y. H. Talmage D. A. Role L. W. (2002). Nicotinic receptor-mediated effects on appetite and food intake. *J. Neurobiol.* 53 618–632. 10.1002/neu.10147 12436425 PMC2367209

[B55] Jones B. H. Kim J. H. Zemel M. B. Woychik H. P. Michaud E. J. Wilkison W. (1996). Upregulation of adipocyte metabolism by agouti protein: possible paracrine actions in obesity of the yellow mouse. *Am. J. Physiol.* 270 E190–E192. 10.1152/ajpendo.1996.270.1.E192 8772492

[B56] Kadowaki T. Yamauchi T. (2005). Adiponectin and adiponectin receptors. *Endocr. Rev.* 26 439–451. 10.1210/er.2005-0005 15897298

[B57] Karschin A. Brockhaus J. Ballanyi K. (1998). KATP channel formation by the sulphonylurea receptors SUR1 with Kir6.2 sub-units in rat dorsal vagal neurons in situ. *J. Physiol.* 509 339–346. 10.1111/j.1469-7793.1998.339bn.x 9575284 PMC2230984

[B58] Kaufhold A. Nigam P. K. Dhir R. N. Shapiro B. H. (2002). Prevention of latently expressed CYP2C11, CYP3A2, and growth hormone defects in neonatally monosodium glutamate-treated male rats by the N-methyl-D-aspartate receptor antagonist dizocilpine maleate. *J. Pharmacol. Exp. Ther.* 302 490–496. 10.1124/jpet.102.034785 12130706

[B59] Kawabata F. Inoue N. Yazawa S. Kawada T. Inoue K. Fushiki T. (2006). Effects of CH-19 sweet, a non-pungent cultivar of red pepper, in decreasing the body weight and suppressing body fat accumulation by sympathetic nerve activation in humans. *Biosci. Biotechnol. Biochem.* 70 2824–2835. 10.1271/bbb.60206 17151481

[B60] Kim J. (2008). Association of CHRNA2 polymorphisms with overweight/obesity and clinical characteristics in a Korean population. *Clin. Chem. Lab. Med.* 46 1085–1089. 10.1515/CCLM.2008.230 18588430

[B61] Kim J. H. Mynatt R. L. Moore J. W. Woychik R. P. Moustaid N. Zemel M. B. (1996). The effects of calcium channel blockade on agouti-induced obesity. *FASEB J.* 10 1646–1652. 9002558

[B62] Kim S. H. Lee Y. M. Jee S. H. Nam C. M. (2003). Effect of sibutramine on weight loss and blood pressure: a meta-analysis of controlled trials. *Obes. Res.* 11 1116–1123. 10.1038/oby.2003.152 12972682

[B63] Klein S. (1999). The war against obesity: attacking a new front. *Am. J. Clin. Nutr.* 69 1061–1063. 10357721 10.1093/ajcn/69.6.1061

[B64] Klockener T. Hess S. Belgardt B. F. Paeger L. Verhagen L. A. Husch A. (2011). High-fat feeding promotes obesity via insulin receptor/PI3K-dependent inhibition of SF-1 VMH neurons. *Nat. Neurosci.* 14 911–918. 10.1038/nn.2847 21642975 PMC3371271

[B65] Kohrt W. M. (1998). Preliminary evidence that DEXA provides an accurate assessment of body composition. *J. Appl. Physiol.* 84 372–377. 9451659 10.1152/jappl.1998.84.1.372

[B66] Kushner R. F. Schoeller D. A. (1986). Estimation of total body water by bioelectrical impedance analysis. *Am. J. Clin. Nutr.* 44 417–424. 3529918 10.1093/ajcn/44.3.417

[B67] Kusudo T. Wang Z. Mizuno A. Suzuki M. Yamashita H. (2012). TRPV4 deficiency increases skeletal muscle metabolic capacity and resistance against diet-induced obesity. *J. Appl. Physiol.* 112 1223–1232. 10.1152/japplphysiol.01070.2011 22207724

[B68] Li G. R. Deng X. L. Sun H. Chung S. S. Tse H. F. Lau C. P. (2006). Ion channels in mesenchymal stem cells from rat bone marrow. *Stem Cells* 24 1519–1528. 10.1634/stemcells.2005-0307 16484345

[B69] Li S. Maude-Griffin R. Pullan A. J. Chen J. D. Z. (2013). Gastric emptying and Ca2+ and K+ channels of circular smooth muscle cells in diet-induced obese prone and resistant rats. *Obesity* 21 326–335. 10.1002/oby.20021 23404843

[B70] Lindsay R. S. Funahashi T. Hanson R. L. Matsuzawa Y. Tanaka S. Tataranni P. A. (2002). Adiponectin and development of type 2 diabetes in the Pima Indian population. *Lancet* 360 57–58. 10.1016/S0140-6736(02)09335-2 12114044

[B71] Liu P. B. Shah P. Croasdell S. Gilbertson T. A. (2011). Transient receptor potential channel type M5 is essential for fat taste. *J. Neurosci.* 31 8634–8642. 10.1523/JNEUROSCI.6273-10.2011 21653867 PMC3125678

[B72] Liu R. H. Mizuta M. Matsukura S. (2004). The expression and functional role of nicotinic acetylcholine receptors in rat adipocytes. *J. Pharmacol. Exp. Ther.* 310 52–58. 10.1124/jpet.103.065037 14993259

[B73] Lucero M. T. Pappone P. A. (1989). Voltage-gated potassium channels in brown fat cells. *J. Gen. Physiol.* 93 451–472. 10.1085/jgp.93.3.451 2467964 PMC2216218

[B74] Ludy M. J. Moore G. E. Mattes R. D. (2011). The effects of capsaicin and capsiate on energy balance: critical review and meta-analyses of studies in humans. *Chem. Senses* 37 103–121. 10.1093/chemse/bjr100 22038945 PMC3257466

[B75] Ma S. Yu H. Zhao Z. Luo Z. Chen J. Ni Y. (2012). Activation of the cold-sensing TRPM8 channel triggers UCP1-dependent thermogenesis and prevents obesity. *J. Mol. Cell Biol.* 4 88–96. 10.1093/jmcb/mjs001 22241835

[B76] MacFarlane S. N. Sontheimer H. (2000). Changes in ion channel expression accompany cell cycle progression of spinal cord astrocytes. *Glia* 30 39–48. 10.1002/(SICI)1098-1136(200003)30:1<39::AID-GLIA5>3.0.CO;2-S 10696143

[B77] Maenhaut N. Van de Voorde J. (2011). Regulation of vascular tone by adipocytes. *BMC Med.* 9:25 10.1186/1741-7015-9-25 PMC3069942 21410966

[B78] Marks D. R. Fadool D. A. (2007). Post-synaptic density 95 (PSD-95) affects insulin induced Kv1.3 channel modulation of the olfactory bulb. *J. Neurochem.* 103 1608–1627. 10.1111/j.1471-4159.2007.04870.x 17854350 PMC2667938

[B79] Marrero M. B. Lucas R. Salet C. Hauser T. A. Mazurov A. Lippiello P. M. (2010). An α-7 nicotinic acetylcholine receptor-selective agonist reduces weight gain and metabolic changes in a mouse model of diabetes. *J. Pharmacol. Exp. Ther.* 332 173–180. 10.1124/jpet.109.154633 19786623

[B80] Martinez J. A. (2000). Body-weight regulation: causes of obesity. *Proc. Nutr. Soc.* 59 337–345. 10.1017/S0029665100000380 10997649

[B81] McArdle W. D. Katch F. I. Katch V. L. (2008). *Fisiologia do Exercício: Energia, Nutrição e Desempenho Humano*, 6 Edn Rio de Janeiro: Guanabara Koogan.

[B82] Miki T. Liss B. Minami K. Shiuchi T. Saraya A. Kashima Y. (2001). ATP-sensitive K+ channels in the hypothalamus are essential for the maintenance of glucose homeostasis. *Nat. Neurosci.* 4 507–512. 10.1038/87455 11319559

[B83] Miller C. W. Casimir D. A. Ntambi J. M. (1996). The mechanism of inhibition of 3T3–L1 preadipocyte differentiation by prostaglandin F2alpha. *Endocrinology* 137 5641–5650. 10.1210/endo.137.12.8940395 8940395

[B84] Mineur Y. S. Abizaid A. Rao Y. Salas R. DiLeone R. J. Gündisch D. (2011). Nicotine decreases food intake through activation of POMC neurons. *Science* 332 1330–1332. 10.1126/science.1201889 21659607 PMC3113664

[B85] Motter A. L. Ahern G. P. (2008). TRPV1-null mice are protected from diet-induced obesity. *FEBS Lett.* 582 2257–2262. 10.1016/j.febslet.2008.05.021 18503767 PMC2486372

[B86] National Institutes of Health [NIH] (1991). Gastrointestinal surgery for severe obesity. Consensus development conference panel. *Ann. Intern. Med.* 115 956–961. 10.7326/0003-4819-115-12-956 1952493

[B87] National Institutes of Health [NIH] (2000). *The Practical Guide: Identification, Evaluation, and Treatment of Overweight and Obesity in Adults.* Bethesda, MD: US Department of Health and Human Services, Public Heatlh Service, National Institutes of Health, National Heart, Lung, and Blood Institute.

[B88] Neal J. W. Clipstone N. A. (2002). Calcineurin mediates the calcium-dependent inhibition of adipocyte differentiation in 3T3-L1 cells. *J. Biol. Chem.* 277 49776–49781. 10.1074/jbc.M207913200 12351639

[B89] Nilius B. Droogmans G. (2001). channels and their functional role in vascular endothelium. *Physiol. Rev.* 81 1415–1459. 10.1016/b978-012656975-9/50030-4 11581493

[B90] Noll J. G. Zeller M. H. Trickett P. K. Putnam F. W. (2007). Obesity risk for female victims of childhood sexual abuse: a prospective study. *Pediatrics* 120 61–67. 10.1542/peds.2006-3058 17606550

[B91] Nozawa K. Kawabata-Shoda E. Doihara H. Kojima R. Okada H. Mochizuki S. (2009). TRPA1 regulates gastrointestinal motility through serotonin release from enterochromaffin cells. *Proc. Natl. Acad. Sci. U.S.A.* 106 3408–3413. 10.1073/pnas.0805323106 19211797 PMC2651261

[B92] Ohashi K. Shibata R. Murohara T. Ouchi N. (2014). Role of anti-inflammatory adipokines in obesity-related diseases. *Trends Endocrinol. Metab.* 25 348–355. 10.1016/j.tem.2014.03.009 24746980

[B93] Oike H. Wakamori M. Mori Y. Nakanishi H. Taguchi R. Misaka T. (2006). Arachidonic acid can function as a signaling modulator by activating the TRPM5 cation channel in taste receptor cells. *Biochim. Biophys. Acta* 1761 1078–1084. 10.1016/j.bbalip.2006.07.005 16935556

[B94] Olds W. H. Xu T. (2014). Regulation of food intake by mechanosensory ion channels in enteric neurons. *Elife* 3 1–14. 10.7554/eLife.04402 PMC4225495 25285450

[B95] Pappone P. A. Ortiz-Miranda S. I. (1993). Blockers of voltage-gated K channels inhibit proliferation of cultured brown fat cells. *Am. J. Physiol. Cell Physiol.* 264 C1014–C1019. 10.1152/ajpcell.1993.264.4.C1014 8476010

[B96] Pereira L. O. Francischi R. P. Lancha A. H. Jr. (2003). Obesidade: hábitos nutricionais, sedentarismo e resistência à insulina. *Arq. Bras. Endocrinol. Metabol.* 47 111–127. 10.1590/S0004-27302003000200003

[B97] Pérez C. A. Huang L. Rong M. Kozak J. A. Preuss A. K. Zhang H. (2002). A transient receptor potential channel expressed in taste receptor cells. *Nat. Neurosci.* 5 1169–1176. 10.1038/nn952 12368808

[B98] Perkins K. A. (1993). Weight gain following smoking cessation. *J. Consult. Clin. Psychol.* 61 768-777. 10.1037/0022-006X.61.5.768 8245274

[B99] Pischon T. Boeing H. Hoffmann K. Bergmann M. Schulze M. B. Overvad K. (2008). General and abdominal adiposity and risk of death in Europe. *N. Engl. J. Med.* 359 2105–2120. 10.1056/NEJMx100031 19005195

[B100] Racette S. B. Deusinger S. S. Robert H. (2003). Obesity: overview of prevalence, etiology and treatment. *Phys. Ther.* 83 276–288. 12620091

[B101] Ramirez-Ponce M. P. Mateos J. C. Bellido J. A. (2003). Human adipose cells have voltage-dependent potassium currents. *J. Membr. Biol.* 196 129–134. 14724749 10.1007/s00232-003-0631-1

[B102] Ramirez-Ponce M. P. Mateos J. C. Carrion N. Bellido J. A. (1996). Voltage-dependent potassium channels in white adipocytes. *Biochem. Biophys. Res. Commun.* 223 250–256. 10.1006/bbrc.1996.0880 8670268

[B103] Ran J. Hirano T. Fukui T. Saito K. Kageyama H. Okada K. (2006). Angiotensin II infusion decreases plasma adiponectin level via its type 1 receptor in rats: an implication for hypertension-related insulin resistance. *Metabolism* 55 478–488. 10.1016/j.metabol.2005.10.00 16546478

[B104] Rosenbaum M. Leibel R. L. Hirsch J. (1997). Obesity. *N. Engl. J. Med.* 337 396–407. 10.1056/NEJM199708073370606 9241130

[B105] Rowe I. C. Boden P. R. Ashford M. L. (1996). Potassium channel dysfunction in hypothalamic glucose-receptive neurons of obese Zucker rats. *J. Physiol.* 497 365–377. 10.1113/jphysiol.1996.sp021774 8961181 PMC1160990

[B106] Russ U. Ringer T. Siemen D. (1993). A voltage-dependent and a voltage-independent potassium channel in brown adipocytes of the rat. *Biochem. Biophys. Acta* 1153 249–256. 10.1016/0005-2736(93)90412-S 8274494

[B107] Satapathy S. K. Ochani M. Dancho M. Hudson L. K. Rosas-Ballina M. Valdes-Ferrer S. I. (2011). Galantamine alleviates inflammation and other obesity-associated complications in high-fat diet-fed mice. *Mol. Med.* 17 599–606. 10.2119/molmed.2011.00083 21738953 PMC3146607

[B108] Scherer P. E. Williams S. Fogliano M. Baldini G. Lodish H. F. (1995). A novel serum protein similar to C1q, produced exclusively in adipocytes. *J. Biol. Chem.* 270 26746–26749. 10.1074/jbc.270.45.26746 7592907

[B109] Schoeller D. A. Van Santen E. Petersen D. W. Dietz W. Jaspan J. Klein P. D. (1980). Total body water measurement in humans with 18O and 2H labeled water. *Am. J. Clin. Nutr.* 33 2686–2693. 6776801 10.1093/ajcn/33.12.2686

[B110] Schwartz M. W. Woods S. C. Porte D. Jr. Seeley R. J. Baskin D. G. (2000). Central nervous system control of food intake. *Nature* 404 661–671. 10.1038/35007534 10766253

[B111] Sclafani A. Springer D. (1976). Dietary obesity in adult rats: similarities to hypothalamic and human obesity syndromes. *Physiol. Behav.* 17 461–471. 10.1016/0031-9384(76)90109-8 1013192

[B112] Sclafani A. Zukerman S. Glendinning J. I. Margolskee R. F. (2007). Fat and carbohydrate preferences in mice. The contribution of alpha-gustducin and Trpm5 taste signaling proteins. *Am. J. Physiol. Regul. Integr. Comp. Physiol.* 293 R1504–R1513. 10.1152/ajpregu.00364.2007 17652359 PMC2375390

[B113] Singh G. M. Danaei G. Farzadfar F. Stevens G. A. Woodward M. Wormser D. (2013). Asia-Pacific Cohort Studies Collaboration (APCSC), and the Diabetes Epidemiology: Collaborative analysis of Diagnostic criteria in Europe (DECODE), and the Emerging Risk Factor Collaboration (ERFC), and the Prospective Studies Collaboration (PSC). The age-specific quantitative effects of metabolic risk factors on cardiovascular diseases and diabetes: a pooled analysis. *PLoS ONE* 8:e65174 10.1371/journal.pone.0065174 PMC3728292 23935815

[B114] Sinha M. K. Caro J. F. (1998). Clinical aspects of leptin. *Vitam. Horm.* 54 1–30. 10.1016/S0083-6729(08)60919-X 9529971

[B115] Snitker S. Fujishima Y. Shen H. Ott S. Pi-Sunyer X. Furuhata Y. (2009). Effects of novel capsinoid treatment on fatness and energy metabolism in humans: possible pharmacogenetic implications. *Am. J. Clin. Nutr.* 89 45–50. 10.3945/ajcn.2008.26561 19056576 PMC3151435

[B116] Sohn J. W. (2013). Ion channels in the central regulation of energy and glucose homeostasis. *Front. Neurosci.* 7:85 10.3389/fnins.2013.00085 PMC3661948 23734095

[B117] Somm E. Guèrardel A. Maouche K. Toulotte A. Veyrat-Durebex C. Rohner-Jeanrenaud F. (2014). Concomitant alpha7 and beta2 nicotinic AChR subunit deficiency leads to impaired energy homeostasis and increased physical activity in mice. *Mol. Genet. Metab.* 112 64–72. 10.1016/j.ymgme.2014.03.003 24685552

[B118] Sukumar P. Sedo A. Li J. Wilson L. A. O’Regan D. Lippiat J. D. (2012). Constitutively active TRPC channels of adipocytes confer a mechanism for sensing dietary fatty acids and regulating adiponectin. *Circ. Res.* 111 191–200. 10.1161/CIRCRESAHA.112.270751 22668831 PMC3605801

[B119] Tallam L. S. Stec D. E. Willis M. A. Silva A. A. Hall J. E. (2005). Melanocortin-4 receptor-deficient mice are not hypertensive or salt-sensitive despite obesity, hyperinsulinemia, and hyperleptinemia. *Hypertension* 46 326–332. 10.1161/01.HYP.0000175474.99326.bf 16027245

[B120] Tam C. S. Lecoultre V. Ravussin E. (2012). Brown adipose tissue: mechanisms and potential therapeutic targets. *Circulation* 125 2782–2791. 10.1161/CIRCULATIONAHA.111.042929 22665886

[B121] Tamura Y. Iwasaki Y. Narukawa M. Watanabe T. (2012). Ingestion of cinnamaldehyde, a TRPA1 agonist, reduces visceral fats in mice fed a high-fat and high-sucrose diet. *J. Nutr. Sci. Vitaminol.* 58 9–13. 10.3177/jnsv.58.9 23007061

[B122] Thaler J. P. Guyenet S. J. Dorfman M. D. Wisse B. E. Schwartz M. W. (2013). Hypothalamic inflammation: marker or mechanism of obesity pathogenesis? *Diabetes* 62 2629–2634. 10.2337/db12-1605 23881189 PMC3717869

[B123] Tong Q. Ye C. Jones J. E. Elmquist J. K. Lowell B. B. (2008). Synaptic release of GABA by AgRP neurons is required for normal regulation of energy balance. *Nat. Neurosci.* 11 998–1000. 10.1038/nn.2167 19160495 PMC2662585

[B124] Tosetti C. Corinaldesi R. Stanghellini V. Pasquali R. Corbelli C. Zoccoli G. (1996). Gastric emptying of solids in morbid obesity. *Int. J. Obes. Relat. Metab. Disord.* 20 200–205. 8653139

[B125] Tschop M. Smiley D. L. Heiman M. L. (2000). Ghrelin induces adiposity in rodents. *Nature* 407 908–913. 10.1038/35038090 11057670

[B126] Tschop M. Wawarta R. Riepl R. L. Friedrich S. Bidlingmaier M. Landgraf R. (2001). Post-prandial decrease of circulating human ghrelin levels. *J. Endocrinol. Invest.* 24 RC19–RC21. 10.1007/BF03351037 11434675

[B127] Tucker K. Overton J. M. Fadool D. A. (2008). Kv1.3 gene-targeted deletion alters longevity and reduces adiposity by increasing locomotion and metabolism in melanocortin-4 receptor-null mice. *Int. J. Obes. (Lond.)* 32 1222–1232. 10.1038/ijo.2008.77 18542083 PMC2737548

[B128] Tucker K. Overton J. M. Fadool D. A. (2012). Diet-induced obesity resistance of Kv1.3-/- mice is olfactory bulb dependent. *J. Neuroendocrinol.* 24 1087-1095. 10.1111/j.1365-2826.2012.02314.x PMC3391345 22435906

[B129] Tucker K. R. Godbey S. J. Thiebaud N. Fadool D. A. (2012). Olfactory ability and object memory in three mouse models of varying body weight, metabolic hormones, and adiposity. *Physiol. Behav.* 107 424–432. 10.1016/j.physbeh.2012.09.007 22995978 PMC3513555

[B130] Uebele V. N. Gotter A. L. Nuss C. E. Kraus R. L. Doran S. M. Garson S. L. (2009). Antagonism of T-type calcium channels inhibits high-fat diet-induced weight gain in mice. *J. Clin. Invest.* 119 1659–1667. 10.1172/JCI36954 19451696 PMC2689113

[B131] Vaisse C. Clement K. Guy-Grand B. Froguel P. (1998). A frameshift mutation in human MC4R is associated with a dominant form of obesity. *Nat. Genet.* 20 113–114. 10.1038/2407 9771699

[B132] Van Gaal L. F. Mertens I. L. De Block C. E. (2006). Mechanisms linking obesity with cardiovascular disease. *Nature* 444 875–880. 10.1038/nature05487 17167476

[B133] Vendrell J. Broch M. Vilarrasa N. Molina A. Gómez J. M. Gutiérrez C. (2004). Resistin, adiponectin, ghrelin, leptin, and proinflammatory cytokines: relationships in obesity. *Obes. Res.* 12 962–971. 10.1038/oby.2004.118 15229336

[B134] Vogler O. Lopez-Bellan A. Alemany R. Tofé S. González M. Quevedo J. (2008). Structure-effect relation of C18 long-chain fatty acids in the reduction of body weight in rats. *Int. J. Obes.* 32 464–473. 10.1038/sj.ijo.0803768 18059405

[B135] Vong L. Ye C. Yang Z. Choi B. Chua-Jr S. Lowell B. B. (2011). Leptin action on GABAergic neurons prevents obesity and reduces inhibitory tone to POMC neurons. *Neuron* 71 142–154. 10.1016/j.neuron.2011.05.028 21745644 PMC3134797

[B136] Vucenik I. Stains J. P. (2012). Obesity and cancer risk: evidence, mechanisms and recommendations. *Ann. N. Y. Acad. Sci.* 1271 37–43. 10.1111/j.1749-6632.2012.06750.x 23050962 PMC3476838

[B137] Whitlock G. Lewington S. Sherliker P. Clarke R. Emberson J. Halsey J. (2009). Body-mass index and cause-specific mortality in 900 000 adults: collaborative analyses of 57 prospective studies. *Lancet* 373 1083–1096. 10.1016/S0140-6736(09)60318-4 19299006 PMC2662372

[B138] Wiedmer P. Nogueiras R. Broglio F. D’Alessio D. Tschöp M. H. (2007). Ghrelin, obesity and diabetes. *Nat. Clin. Pract. Endocrinol. Metab.* 3 705–712. 10.1038/ncpendmet0625 17893689

[B139] Williams D. L. Schwartz M. W. (2005). The melanocortin system as a central integrator of direct and indirect controls of food intake. *Am. J. Physiol. Regul. Integr. Comp. Physiol.* 289 R2–R3. 10.1152/ajpregu.00226.2005 15956761

[B140] Williams K. W. Margatho L. O. Lee C. E. Choi M. Lee S. Scott M. M. (2010). Segregation of acute leptin and insulin effects in distinct populations of arcuate proopiomelanocortin neurons. *J. Neurosci.* 30 2472–2479. 10.1523/JNEUROSCI.3118-09.2010 20164331 PMC2836776

[B141] Williams K. W. Scott M. M. Elmquist J. K. (2011a). Modulation of the central melanocortin system by leptin, insulin, and serotonin: coordinated actions in a dispersed neuronal network. *Eur. J. Pharmacol.* 660 2–12. 10.1016/j.cmet.2009.09.008 21211525 PMC3085544

[B142] Williams K. W. Sohn J. W. Donato J. Jr. Lee C. E. Zhao J. J. Elmquist J. K. (2011b). The acute effects of leptin require PI3K signaling in the hypothalamic ventral premammillary nucleus. *J. Neurosci.* 31 13147–13156. 10.1523/JNEUROSCI.2602-11.2011 21917798 PMC3319415

[B143] World Health Organization [WHO] (2014). *Obesity: Situation and Trends.* Available at: http://www.who.int/gho/ncd/risk_factors/obesity_text/en/ (accessed October 2014).

[B144] Wright R. A. Krinsky S. Fleeman C. Trujillo J. Teague E. (1983). Gastric emptying and obesity. *Gastroenterology* 84 747–751. 6825986

[B145] Wu Q. Boyle M. P. Palmiter R. D. (2009). Loss of GABAergic signaling by AgRP neurons to the parabrachial nucleus leads to starvation. *Cell* 137 1225–1234. 10.1016/j.cell.2009.04.022 19563755 PMC2729323

[B146] Xu J. Koni P. A. Wang P. Li G. Kaczmarek L. K. Wu Y. (2003). The voltage-gated potassium channel Kv1.3 regulates energy homeostasis and body weight. *Hum. Mol. Genet.* 12 551–559. 10.1093/hmg/ddg049 12588802

[B147] Xu J. Wang P. Li Y. Li G. Kaczmarek L. K. Wu Y. (2004). The voltage-gated potassium channel Kv1.3 regulates peripheral insulin senstivitiy. *Proc. Natl. Acad. Sci. U.S.A.* 101 3112–3117. 10.1073/pnas.0308450100 14981264 PMC365752

[B148] Yamauchi T. Kamon J. Waki H. Terauchi Y. Kubota N. Hara K. (2001). The fat-derived hormone adiponectin reverses insulin resistance associated with both lipoatrophy and obesity. *Nat. Med.* 7 941–946. 10.1038/90984 11479627

[B149] Yang S. B. Tien A. C. Boddupalli G. Xu A. W. Jan Y. N. Jan L. Y. (2012). Rapamycin ameliorates age-dependent obesity associated with increased mTOR signaling in hypothalamic POMC neurons. *Neuron* 75 425–436. 10.1016/j.neuron.2012.03.043 22884327 PMC3467009

[B150] Ye L. Kleiner S. Wu J. Sah R. Gupta R. K. Banks A. S. (2012). TRPV4 is a regulator of adipose oxidative metabolism, inflammation, and energy homeostasis. *Cell* 151 96–110. 10.1016/j.cell.2012.08.034 23021218 PMC3477522

[B151] Yoshida T. Umekawa T. Wakabayashi Y. Sakane N. Kondo M. (1994). Mechanism of anti-obesity action of benidipine hydrochloride in mice. *Int. J. Obes. Relat. Metab. Disord.* 18 776–779. 7866480

[B152] Yoshioka M. St-Pierre S. Drapeau V. Dionne I. Doucet E. Suzuki M. (1999). Effects of red pepper on appetite and energy intake. *Br. J. Nutr.* 82 115–123. 10.1017/S0007114599001269 10743483

[B153] Zahorska-Markiewicz B. Jonderko K. Lelek A. Skrzypek D. (1986). Gastric emptying in obesity. *Hum. Nutr. Clin. Nutr.* 40 309–313. 3744891

[B154] Zaragoza R. M. Lopez M. L. Villaneuva S. L. Ortiz R. A. Villanueva G. L. (2005). Efficacy and safety of slow-release fenproporex for the treatment of obesity. *Rev. Mex. Cardiol.* 16 146–154.

[B155] Zawar C. Plant T. D. Schirra C. Konnerth A. Neumcke B. (1999). Cell-type specific expression of ATP-sensitive potassium channels in the rat hippocampus. *J. Physiol.* 514 327–341. 10.1111/j.1469-7793.1999.315ae.x 9852317 PMC2269073

[B156] Zemel M. B. Kim J. H. Woychik H. P. Michaud E. J. Kadwell S. H. Patel I. R. (1995). Agouti regulation of intracellular calcium: role in the insulin resistance of viable yellow mice. *Proc. Nail. Acad. Sci. U.S.A.* 92 4733–4737. 10.1073/pnas.92.11.4733 PMC41781 7761392

[B157] Zhang L. L. Liu D. Y. Ma L. Q. Luo Z. D. Cao T. B. Zhong J. (2007). Activation of transient receptor potential vanilloid type-1 channel prevents adipogenesis and obesity. *Circ. Res.* 100 1063–1070. 10.1161/01.RES.0000262653.84850.8b 17347480

[B158] Zhang Y. Hoon M. A. Chandrashekar J. Mueller K. L. Cook B. Wu D. (2003). Coding of sweet, bitter, and umami tastes: different receptor cells sharing similar signaling pathways. *Cell* 112 293–301. 10.1016/S0092-8674(03)00071-0 12581520

[B159] Zhang Z. Zhang W. Jung D. Y. Ko H. J. Lee Y. Friedline R. H. (2012). TRPM2 Ca2+ channel regulates energy balance and glucose metabolism. *Am. J. Physiol. Endocrinol. Metab.* 302 E807–E816. 10.1152/ajpendo.00239.2011 22275755 PMC3330711

[B160] Zhu A. Z. X. Renner C. C. Hatsukami D. K. Benowitz N. L. Tyndale R. F. (2013). CHRNA5-A3-B4 genetic variants alter nicotine intake and interact with tobacco use to influence body weight in Alaska Native tobacco users. *Addiction* 108 1818–1828. 10.1111/add.12250 23692359 PMC3775934

[B161] Zhu Y. Yang J. Yeh F. Cole S. A. Haack K. Lee E. T. (2014). Joint association of nicotinic acetylcholine receptor variants with abdominal obesity in american indians: the Strong Heart Family Study. *PLoS ONE* 9:e102220 10.1371/journal.pone.0102220 PMC4103845 25036316

[B162] Zoli M. Picciotto M. R. (2012). Nicotinic regulation of energy homeostasis. *Nicotine Tob. Res.* 14 1270–1290. 10.1093/ntr/nts159 22990212 PMC3611985

